# Simultaneous Fluorescence and Phosphorescence Lifetime Imaging Microscopy in Living Cells

**DOI:** 10.1038/srep14334

**Published:** 2015-09-22

**Authors:** Karolina Jahn, Volker Buschmann, Carsten Hille

**Affiliations:** 1 Physical Chemistry/ALS ComBi, Institute of Chemistry, University of Potsdam, Potsdam, Germany; 2 PicoQuant GmbH, Berlin, Germany

## Abstract

In living cells, there are always a plethora of processes taking place at the same time. Their precise regulation is the basis of cellular functions, since small failures can lead to severe dysfunctions. For a comprehensive understanding of intracellular homeostasis, simultaneous multiparameter detection is a versatile tool for revealing the spatial and temporal interactions of intracellular parameters. Here, a recently developed time-correlated single-photon counting (TCSPC) board was evaluated for simultaneous fluorescence and phosphorescence lifetime imaging microscopy (FLIM/PLIM). Therefore, the metabolic activity in insect salivary glands was investigated by recording *ns*-decaying intrinsic cellular fluorescence, mainly related to oxidized flavin adenine dinucleotide (FAD) and the *μs*-decaying phosphorescence of the oxygen-sensitive ruthenium-complex Kr341. Due to dopamine stimulation, the metabolic activity of salivary glands increased, causing a higher pericellular oxygen consumption and a resulting increase in Kr341 phosphorescence decay time. Furthermore, FAD fluorescence decay time decreased, presumably due to protein binding, thus inducing a quenching of FAD fluorescence decay time. Through application of the metabolic drugs antimycin and FCCP, the recorded signals could be assigned to a mitochondrial origin. The dopamine-induced changes could be observed in sequential FLIM and PLIM recordings, as well as in simultaneous FLIM/PLIM recordings using an intermediate TCSPC timing resolution.

The precise regulation of intracellular homeostasis is the basis of all cellular functions, as small failures can potentially lead to severe dysfunctions. Thus, a comprehensive understanding of intracellular processes is of particular interest. Highly sensitive and non-invasive fluorescence microscopy is a versatile tool for investigating cellular processes^1^. One basic prerequisite therefore is that exogenous or endogenous fluorophores exhibit unique spectroscopic properties and display a specific sensitivity to cellular parameter changes^2^.

In addition to well-established single parameter detection, special novel concepts for simultaneous multiparameter detection can extend our view of cellular machinery. In contrast to sequential data acquisition, simultaneous data acquisition allows faster multiparameter detection and thus the observation of rapid cellular processes. Moreover, cell damage is diminished and this significantly contributes to the analysis of spatial and temporal interactions of cellular parameters. It must be emphasized that the term ‘multiparameter detection’ in this context means the simultaneous observation of at least two physiological parameters by using the appropriate number of analyte-sensitive fluorophores and detecting changes in their fluorescent properties. In this case, each fluorescent sensor can easily be detected using spectral separation *via* different excitation or emission wavelengths. Instead of using several excitation wavelengths for one-photon excitation, the application of two-photon excitation can be advantageous. In comparison to one-photon absorption spectra, two-photon absorption spectra tend to be broader, meaning that spectrally different fluorescent sensors can still be excited by one distinct two-photon excitation wavelength^3^. Unfortunately, the spectral discrimination of fluorescent sensors is often insufficient, as broad absorption or fluorescence spectra cause signal crosstalk to a certain extent. For crosstalk correction, the application of narrow bandpass filters in the emission pathway or a sequential one-photon excitation could be helpful at the cost of sensitivity and acquisition time. Linear unmixing, on the other hand, does not prevent the crosstalk during data acquisition, but engages mathematical concepts for downstream crosstalk correction with the help of proper reference spectra^4^. Regarding analyte quantification, ratiometric imaging enables a reliable determination of analyte concentrations. Here, fluorescent reporters are used which exhibit a shift in their excitation or emission spectra due to cellular parameter changes. Thus, depending on the reporter type, two fluorescence images are recorded using two excitation or emission wavelengths. The calculated ratio allows for correction of varying indicators’ fluorescence intensity due to an uneven indicator concentration or uneven optical pathway^5^.

In addition to the commonly recorded fluorescence intensity, further information could be obtained from the fluorescence decay time (τ)^6^. The fluorescence decay time is a unique, intrinsic property of a fluorescent reporter which shows a sensitivity towards changes in the microenvironment such as physiological parameters in living cells. In contrast to the fluorescence intensity, the decay time is largely independent of the fluorophore concentration, the excitation wavelength, as well as the excitation exposure time. Thereby, artefacts resulting from sensor leakage and photobleaching can be avoided. The environmental sensitivity in combination with its independence of fluorophore concentration makes fluorescence decay time recordings a reasonable method to complement fluorescence intensity recordings^7^. Fluorescence lifetime imaging microscopy (FLIM) uses the fluorescence decay time as the recording parameter, which can then be directly linked to changes in the respective physiological parameter. Among the FLIM techniques, the time-correlated single-photon counting (TCSPC) technique provides the highest time resolution as well as the best decay time accuracy, and photon efficiency^8^. However, reports on the solely time-resolved separation of different fluorescent sensors are limited^9^,^10^. One reason for this is the number of photons required for a reliable analysis of multiexponential fluorescence decay behaviour, which increases dramatically as the number of decay time components increases, leading to longer acquisition times and possible cell damage^11^. Inadequate photon statistics can be used for the analysis of complex decay behaviours, if the contributing components differ significantly in their decay times and possess similar relative amplitudes^12^. However, in complex biological systems, this is generally not the case. Here, a pattern-matching approach could be applied as an alternative^13^.

Another approach is the combination of fluorescence and phosphorescence lifetime imaging (FLIM/PLIM), since it provides access to the discrimination of luminescent sensors with notably separated luminescence decay times. Nevertheless, simultaneous recordings of fluorescent and phosphorescent decay curves are technically challenging, because the time scale differs by several orders of magnitude. In the present study, a recently developed TCSPC board TimeHarp 260 PICO was evaluated for FLIM/PLIM measurements^14^. It provides an exceedingly broad TCSPC time channel resolution range of 25 ps to 52 μs, resulting in a full-scale time window of 819 ns to 1.71 s in the standard mode, which can be even further extended to 171 s by a dedicated “long range mode”^14^. For example, it should be possible to detect simultaneously the *ns*-decaying intrinsic autofluorescence of living cells and the *μs*-decaying phosphorescence of an oxygen-sensitive metal organic complex. The multi-stop functionality and short dead time of the board allows several phosphorescence photons to be captured in a single excitation cycle, which is crucial to limit the necessary acquisition time.

Autofluorescence imaging is a powerful tool for characterising the metabolic state of cells or tissues. It provides information about cell vitality, as in case of anomalies, the cellular metabolism is altered^15^,^16^. Autofluorescence mainly results from the redox pairs nicotinamide adenine dinucleotide (NADH/NAD^+^) and flavin adenine dinucleotide (FADH_2_/FAD), serving as electron carriers during ATP-producing oxidative phosphorylation. Both redox pairs exist in two physiological forms, free and protein-bound forms. Upon binding to mitochondrial proteins, these redox pairs are associated with ATP production so that monitoring of the free/bound ratio can provide information about the metabolic state. Though, only reduced NADH and oxidized FAD can be monitored with fluorescence microscopy, as their respective redox partners are non-fluorescent. The fluorescence spectra of free and protein-bound forms differ only slightly, making them unfavourable for fluorescence intensity measurements^17^. Here, further information about the metabolic state can be obtained from FLIM recordings, since free and protein-bound forms have well separated fluorescence decay times. For instance, the fluorescence decay time of FAD decreases upon protein binding, whereas that of NADH increases upon protein binding^17^. In addition, molecular oxygen acts as a final electron acceptor in the electron transport chain, so that changes in oxygen concentration also reflect cellular activity^18^. For investigating molecular oxygen in living tissue, mainly metal organic complexes are used, whose slowly decaying luminescence is dynamically quenched by triplet oxygen ^3^O_2_, making them favourable for PLIM recordings^19^,^20^. Here, the oxygen-sensitive signal can easily be separate from the faster decaying autofluorescence signal.

In the present study, pericellular oxygen in American cockroach salivary glands was recorded using the *μs*-decaying ruthenium-based complex Kr341^21^, combined with simultaneous intracellular FAD fluorescence recordings. Two parameters could therefore be recorded simultaneously in a single FLIM/PLIM measurement. Cockroach salivary glands are a well-established model system for biogenic amine induced transepithelial ion transport processes^22^. Since the biogenic amine dopamine induces saliva secretion and thus maintains increased metabolic activity, expected opposing dopamine induced changes in oxygen and FAD could be unravelled.

## Results and Discussion

### *Time-resolved luminescence recordings of O*_*2*_

**Figure 1:**
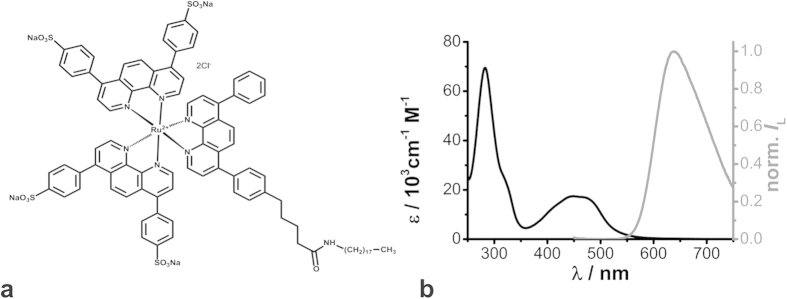
Steady-state absorption and fluorescence measurements of Kr341 *in vitro*. (**a**) Chemical structure of the O_2_-sensitive metal organic complex Kr341^18^. (**b**) Absorption (black) and luminescence emission (grey) spectra of Kr341 in oxygen-equilibrated aqueous solution.

For *in situ* O_2_ detection, the metal organic complex Kr341 was used, composed of three phenantroline ligands and ruthenium as the central ion (Fig. 1a)^21^. Thus, its chemical structure is similar to other well-characterised commercial O_2_ sensor dyes^23^,^24^. In aqueous solution, Kr341 exhibited two absorption maxima (λ_abs_ = 283 nm and λ_abs_ = 449 nm) and a broad emission band around λ_em_ = 638 nm (Fig. 1b).

The luminescence emitted from this metal organic complex can be dynamically quenched by triplet oxygen ^3^O_2_ *via* triplet-triplet annihilation^25^. The singlet oxygen ^1^O_2_ formed is harmful for cells, and therefore needs to be captured by antifading agents, such as DABCO^26^. The dependence of the measured luminescence decay time τ on the O_2_ concentration [O_2_] is described by the Stern-Volmer equation (equation 1)^23^.


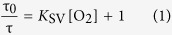


Here, τ_0_ corresponds to the luminescence decay time in the absence of O_2_ and *K*_SV_ is the Stern-Volmer constant, indicating the O_2_-sensitivity of the Kr341 luminescence. In aqueous solution, Kr341 exhibited values of τ_0_ = (2.08 ± 0.05) μs and *K*_SV_ = (7 ± 0.3) × 10^−4^ μM^−1^, respectively (see Supplementary Fig. S1). These values are slightly lower than previously published data for analogue O_2_ sensor dyes, probably due to structural differences in the dye scaffolds^27^,^28^.

**Figure 2:**
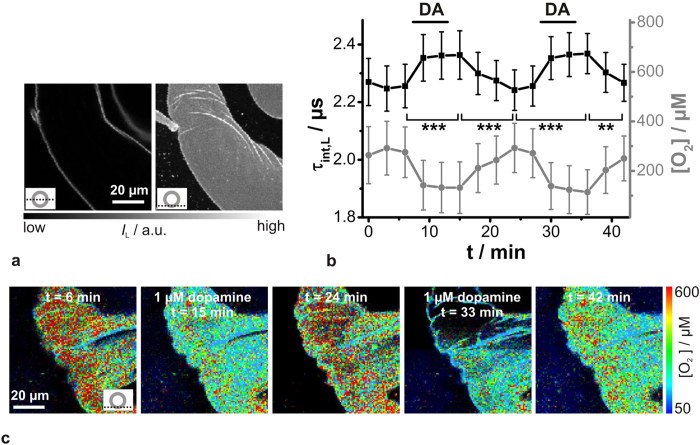
Analysis of dopamine-induced [O_2_] changes in Kr341-treated salivary duct cells by PLIM recordings. (**a**) Luminescence intensity *I*_L_ images of Kr341-treated salivary duct cells. The optical section plane through the gland duct is indicated (lower left of each image). (**b**) 1 μM dopamine-induced (DA) changes in Kr341 luminescence decay time τ_int,L_ (black data points; means ± SEM, *N* = 12) and statistical analyses (***P < 0.001, **P < 0.01) and corresponding pericellular [O_2_] values (grey data points) based on a three-point *in situ* calibration procedure. (**c**) Representative PLIM images of the Kr341-treated ducts at distinct time points. Recording parameters: 200 pixel × 200 pixel, 80 μm × 80 μm, pixel dwell time 0.4 ms/pixel, TCSPC time resolution 800 ps, repetition rate 50 kHz. Warmer colours indicate higher pericellular [O_2_] values and shorter luminescence decay times and cooler colours *vice versa*. The optical section plane through the gland duct is indicated in the lower right of the first image. The luminescence intensity levels of the PLIM images were adjusted for better visibility.

For *in situ* investigations of molecular oxygen, salivary glands were incubated with Kr341 and, subsequently, luminescence intensity *I*_L_ images of the duct region were recorded. Images clearly depicted an exclusive accumulation of Kr341 in the basolateral membrane of the duct cells, whereas almost no Kr341 luminescence could be observed in the intracellular region (Fig. 2a). This behaviour could be attributed to the dye structure containing a long hydrophobic hydrocarbon chain and has also been described for structural analogues of membrane-associated sensor dyes^29^. The restricted localisation of Kr341 in the living tissue is beneficial for physiological studies, as harmful ^1^O_2_ formed by the dynamic quenching of Kr341 with ^3^O_2_ can interact with biological tissue only in a small, limited membrane region and is continuously washed out by the surrounding buffer solution. The exclusive localisation of Kr341 in the basolateral membrane domain allowed pericellular O_2_ measurements at this distinct surface. Due to tissue respiration the intracellular O_2_ concentration is somewhat lower than the pericellular O_2_ concentration^30^. However, the O_2_ concentration gradient depends on respiration activity. In consideration of the diffusion law, decreasing intracellular O_2_ concentration will lead to lower pericellular O_2_ concentration^30^. Therewith, oxygen-sensors located to the cell membrane are suitable for investigating cellular activity.

For cockroach salivary glands, it is known that the biogenic amine dopamine stimulates saliva secretion, including transepithelial ion-transport processes^22^. Thus, the metabolic activity of acinar peripheral cells for the formation of a primary saliva, as well as of duct cells for saliva modification, is enhanced and thus the intracellular O_2_ consumption as well.

The luminescence decay curves obtained from time-resolved luminescence recordings of Kr341-loaded duct cells displayed a multiexponential decay behaviour. Hence, tail-fitting by means of a biexponential decay function yielded reasonable residuals and reduced 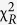 values in comparison to a monoexponential decay function (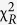 1.23 *vs.* 6.73) (see Supplementary Fig. S2). The application of a triexponential fitting analysis did not significantly improve the quality of the residuals and reduced 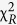 values (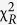 1.23 *vs.* 1.04) (see Supplementary Fig. S2). Thus, all decay curves were analysed using the biexponential fitting model. For further examinations, the corresponding intensity-weighted average luminescence decay times τ_int,L_ were calculated^31^. In the presence of 1 μM dopamine the maximal secretory rate in isolated salivary glands is induced so that τ_int,L_ increased to a statistically significant degree from 2.24 μs to 2.36 μs (P < 0.01) and recovered to resting state after dopamine washout (Fig. 2b)^32^. Here, the salivary gland preparations displayed individual variations (see Supplementary Fig. S5). By performing a simple three-point *in situ* calibration, it is possible to estimate pericellular [O_2_] from the obtained τ_int,L_ values (equation 1 and Fig. 2b)^33^. Therefore, the Kr341-loaded duct cells were perfused with physiological solutions of defined [O_2_], revealing *in situ* calibration parameters τ_int,L,0_ = (2.44 ± 0.04) μs and *K*_SV_ = (3 ± 0.3) × 10^−4^ μM^−1^ (see Supplementary Fig. S1). The *in situ* calibration procedure was challenging, as the perfusion chamber could not be completely sealed off. However, the estimated [O_2_] values based on this simple three-point *in situ* calibration were in good agreement with the [O_2_] values expected for living cells^18^. A similar response to a second dopamine stimulus proved the reversibility of this process and the viability of the duct cells (Fig. 2b). The dopamine behaviour could also be demonstrated by false-colour coded PLIM images, whereby warmer colours corresponded to shorter τ_int,L_ values and higher pericellular [O_2_] values (Fig. 2c).

**Figure 3:**
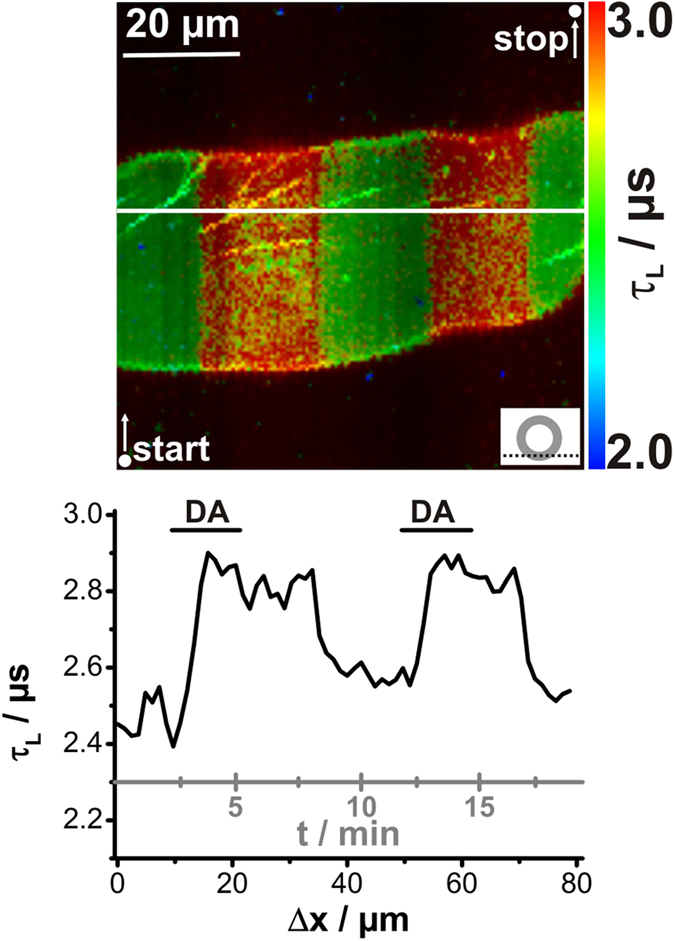
Improved temporal resolution of PLIM recordings of Kr341-treated salivary ducts. Recording parameters: 200 pixel × 200 pixel, 80 μm × 80 μm, pixel dwell time 25 ms/pixel, TCSPC time resolution 800 ps, repetition rate 50 kHz. Bidirectional scanning resulted in a time resolution of approx. 5 s per scanning line. The first and last recorded pixels of the image are indicated by white points and the corresponding white arrows show the scanning direction. During image acquisition, gland preparation was stimulated twice with 1 μM dopamine (DA). The optical section plane through the gland duct is indicated in the lower right. Warmer colours indicate lower pericellular [O_2_] and longer luminescence decay times τ_L_. and cooler colours *vice versa*. The graph below the PLIM image corresponds to the white solid line and depicts τ_L_-values as a function of length Δ*x*.

The chosen data acquisition rate of 0.33 min^−1^ (1 image every 3 minutes) was probably too slow to unravel transient changes in [O_2_] (Fig. 2c). Therefore, a new approach was evaluated by increasing the pixel dwell time significantly from 0.4 ms/pixel to 25 ms/pixel. Thus, recording an image of 80 μm **×** 80 μm with 200 pixel **×** 200 pixel required a total acquisition time of approx. 19 min. During the acquisition of such an image, the salivary gland preparation was stimulated twice with dopamine (Fig. 3). Again, warmer colours corresponded to lower [O_2_] and longer luminescence decay times, and *vice versa*. The PLIM image obtained clearly showed distinct regions of luminescence decay behaviour, as the colour turned reversibly from green to red in the presence or absence of dopamine, respectively. The graph below the PLIM image indicates the luminescence decay time τ_L_ as a function of length Δ× as extracted from the cross-section (white solid line). Thus, a temporal resolution of approx. 5 s could be realized and the behaviour still corresponded to the previous findings (Fig. 2b *vs*. Fig. 3). So, in the presence of dopamine, the luminescence decay time rose rapidly until it reached a plateau, and decreased time-delayed after dopamine washout. In summary, it can be stated that the approach of increasing the image acquisition time offers the possibility of studying rapid transient processes using PLIM. However, for the dopamine-induced [O_2_] changes in the cockroach salivary glands, the temporal resolution of one image per 3 min was sufficient.

### Time-resolved luminescence recordings of FAD

**Figure 4:**
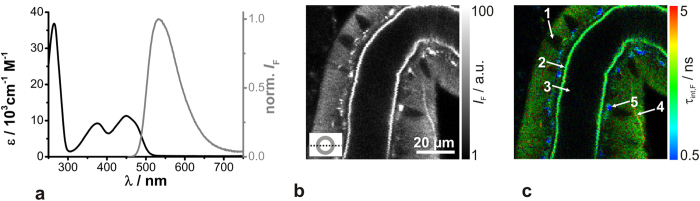
Steady-state absorption and fluorescence measurements of FAD *in vitro* and autofluorescence images of salivary duct cells. (**a**) Absorption (black) and fluorescence emission (grey) spectra of FAD in phosphate buffered saline at pH = 7.0. (**b,c**) Fluorescence intensity image and the corresponding FLIM image of an unstained duct (optical section plane is indicated in the lower left) displaying the autofluorescence after excitation at λ_ex_ = 470 nm, with the following structural features: 1 cell nucleus, 2 luminal cuticle, 3 lumen, 4 trachea, 5 point-shaped structures. Recording parameters: 200 pixel × 200 pixel, 80 μm × 80 μm, pixel dwell time 0.4 ms/pixel, TCSPC time resolution 25 ps, repetition rate 20 MHz.

FADH_2_ and FAD are a redox pair functioning as a potential biomarker for metabolic activities in eukaryotic cells. However, only oxidized FAD is fluorescent and can be monitored without exogenous labelling. In phosphate buffered saline, FAD showed three absorption bands (λ_abs_ = 264 nm, λ_abs_ = 375 nm and λ_abs_ = 450 nm) and a broad emission band (λ_em_ > 520 nm (Fig. 4a)), as known from the literature^34^. In addition, isolated salivary glands without any dye treatment also showed a bright fluorescence emission >500 nm after excitation at λ_ex_ = 470 nm, which could presumably be attributed to FAD fluorescence. Cellular autofluorescence resulting from reduced NADH was not observable under these experimental conditions, since it exhibits absorption and emission maxima around 340 nm and 460 nm, respectively (Fig. 4b,c)^6^. In contrast, two-photon excitation at λ_ex_ = 720–800 nm would lead to excitation of both NADH and FAD. So, for an exclusive 2P-excitation of FAD wavelengths of more than 800 nm are needed^35^,^36^. In addition to the FAD fluorescence, autofluorescence signals could be also obtained from the cuticle lining the ductal lumen and from apically located, point-shaped structures of yet unknown origin (Fig. 4b,c).

**Figure 5:**
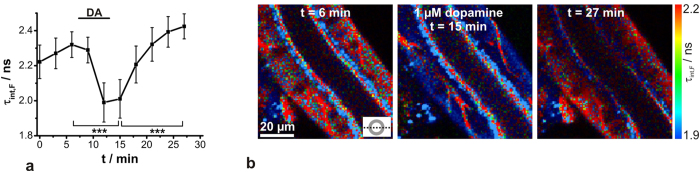
Analysis of dopamine-induced FAD changes in salivary duct cells by FLIM recordings. (**a**) Determination of resting τ_int,F_ (most likely FAD) in cockroach salivary duct cells and the effect of 1 μM dopamine (DA) stimulation analysed from FLIM images; means ± SEM, *N* = 12, statistical analyses (***P < 0.001). (**b**) Representative FLIM images at distinct time points. Recording parameters: 200 pixel × 200 pixel, 80 μm × 80 μm, pixel dwell time 0.4 ms/pixel, TCSPC time resolution 25 ps, repetition rate 20 MHz. The optical section plane through the gland duct is indicated in the lower right. After dopamine stimulation, the autofluorescence intensity signal decreased tremendously, and therefore the fluorescence intensity levels of FLIM images were adjusted for better visibility.

A decrease in the autofluorescence intensity of isolated cockroach salivary glands in the presence of 1 μM dopamine after excitation at 470 nm had been reported previously, which was assumed to be a result of higher metabolic activity^37^. However, this intensity change could be attributed to changes in FAD, as well as to cell volume changes or photobleaching effects. Therefore, the *ns*-decaying behaviour of FAD was studied with FLIM recordings. Free FAD in buffer solution exhibits a mono- or biexponential fluorescence decay behaviour, with average decay times ranging from 2.3 ns to 3.1 ns^16^,^38^,^39^. Although free FAD can be found in living cells, FAD mostly acts as a redox cofactor to proteins called flavoproteins. In contrast to free FAD, protein-bound FAD exhibits much faster and multiexponential fluorescence decay behaviour, with average decay times <0.1 ns^7^,^40^. The fluorescence decay curves obtained from FLIM images of untreated isolated salivary glands showed complex multiexponential decay behaviour (see Supplementary Fig. S3). They could be fitted by means of a biexponential deconvolution fitting model to yield reasonable 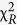
values. However, larger deviations were observed around the fluorescence peak maximum, indicating an additional decay time component (see Supplementary Fig. S3). However, a triexponential fitting model yielded only slightly improved 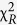 values and residuals (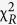 2.53 *vs*. 1.90). In addition, the third decay time component was calculated to approx. 50 ps. This short decay time component was considered uncertain, since such short decay times cannot be reliably resolved with the present setup displaying an IRF of 530 ps (FWHM). So, all decay curves were analysed using a biexponential fitting model. For further analyses, the intensity-weighted average fluorescence decay time τ_int,F_ was calculated. Thereby, it turned out that τ_int,F_ decreased statistically significantly from 2.3 ns to 2.0 ns in the presence of 1 μM dopamine (*P* < 0.01) and recovered to resting state after dopamine washout (Fig. 5a). Here, the salivary gland preparations displayed individual variations (see Supplementary Fig. S5). Stimulation of salivary glands with the biogenic amine dopamine induces saliva secretion, which involves an increase in metabolic activity. In this case, FAD is thought to bind to mitochondrial proteins (e.g. succinate dehydrogenase), which can be directly associated with ATP synthesis in oxidative phosphorylation. However, this protein binding induces a quenching of the FAD fluorescence decay time, as observed during dopamine stimulation. The described behaviour could also be identified using false-colour coded FLIM images (Fig. 5b). Moreover, from the FLIM images, it could be observed that the autofluorescence intensity of the duct cells decreased reversibly due to dopamine stimulation, additionally indicating a quenching of FAD fluorescence. In contrast, the autofluorescence arising from the luminal cuticle and trachea did not change during dopamine stimulation.

### Simultaneous O_*2*_ *and FAD recordings in cockroach salivary ducts*

**Figure 6:**
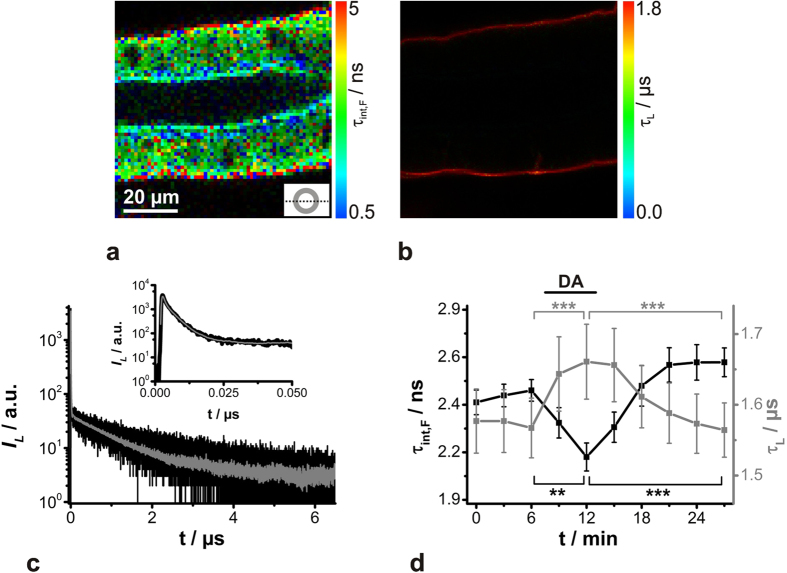
Observation of dopamine-induced (DA) changes of O_2_ and FAD in cockroach salivary duct cells by simultaneous PLIM/FLIM recordings. Gland preparations were incubated with the O_2_-sensitive sensor Kr341 and, subsequently, its luminescence was recorded together with intrinsic autofluorescence by choosing an intermediate TCSPC time resolution. Representative (**a**) FLIM and (**b**) corresponding PLIM images of a Kr341-treated salivary duct are shown. Recording parameters: 200 pixel × 200 pixel, 80 μm × 80 μm, pixel dwell time 2.0 ms/pixel, TCSPC time resolution 200 ps, repetition rate 50 kHz. The optical section plane through the gland duct is indicated in the lower right. (**c**) Luminescence decay curve (black) of simultaneously recorded *ns*-decaying FAD and *μs*-decaying O_2_ sensor Kr341 and the corresponding fits (grey solid line; for details, see Fig S4A,B). The inset depicts an enlargement of the steeply falling fluorescence at the beginning of the decay curve. (**d**) Simultaneous determination of resting τ_int,F_ (black, FAD) and τ_L_ (grey, O_2_) in cockroach salivary duct cells and the effect of dopamine stimulation analysed from PLIM/FLIM images; means ± SEM of *N* = 10 and statistical analyses (***P < 0.001, **P < 0.01).

In order to understand the complex cellular machinery more precisely, simultaneous analysis of the spatio-temporal behaviour of several physiological parameters is mandatory. By means of the novel TCSPC board TimeHarp 260, it was generally possible to record *ns*-decaying FAD and *μs*-decaying O_2_ sensor simultaneously. The time resolution of the 32768 histogram channels was set to an intermediate TCSPC time resolution of 200 ps, yielding a full-scale time range of 6.55 μs. Indeed, under these conditions, neither the *ns*-decay regime nor the *μs*-decay regime can be analysed exactly, but the time resolution is still acceptable for observing physiologically relevant relative signal changes. Thus, isolated salivary glands were incubated with Kr341, and its luminescence was subsequently recorded together with the intrinsic autofluorescence. Analysis of the luminescence decay time components clearly indicated a spatial separation of an intracellular *ns*-decaying FAD signal and a *μs*-decaying Kr341 signal at the basolateral membrane (Fig. 6a,b). The obtained luminescence decay curves extracted from PLIM/FLIM images showed two distinguishable extremes of luminescence decay behaviour, an initial steeply falling fluorescence decay and a subsequent, moderately falling luminescence decay (Fig. 6c). The part of the decay curve which could mainly be attributed to the fluorescence (*t* ~ 0–40 ns) was fitted by a biexponential deconvolution fitting model (see Supplementary Fig. S4). The remaining part (*t* > 60 ns) was fitted by a monoexponential tail-fit, yielding satisfactory 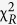 values (see Supplementary Fig. S4). Finally, the changes in these calculated luminescence decay times during 1 μM dopamine stimulation are shown in Fig. 6d. The results confirmed the observations found in individual pericellular [O_2_] or FAD recordings. So, in the presence of 1 μM dopamine, τ_int,F_ probably decreased due to boosted protein binding of FAD, whereas, at the same time, τ_L_ increased due to elevated oxygen consumption. Although the available data point and the TCSPC timing resolution from the decay curves were suboptimal, statistically significant dopamine-induced changes in [O_2_] and FAD could be detected simultaneously (*P* < 0.01). Both parameters (τ_int,F_; τ_L_) changed reversibly and indicate a higher metabolic activity during dopamine application.

Besides NADH, mainly FAD serves as an electron carrier during the ATP-producing oxidative phosphorylation, in which oxygen acts as the final electron acceptor in the electron transport chain^18^. Thus, the ratio of oxidized to reduced electron carriers, as well as changes in the oxygen concentration, provide insight into the metabolic state of a living cell. Since only oxidized FAD and reduced NADH are significantly fluorescent, the fluorescence intensity-based FAD/NADH redox ratio has often been used to unravel metabolic activity^41^,^42^. In doing so, a common procedure for assigning those signals to mitochondrial origins and modulating them is the application of metabolic drugs. Here, simultaneous PLIM and FLIM recordings of oxygen and FAD were carried out in order to evaluate whether luminescence decay time changes can also reflect perturbations in cellular metabolism induced by different metabolic drugs.

**Figure 7:**
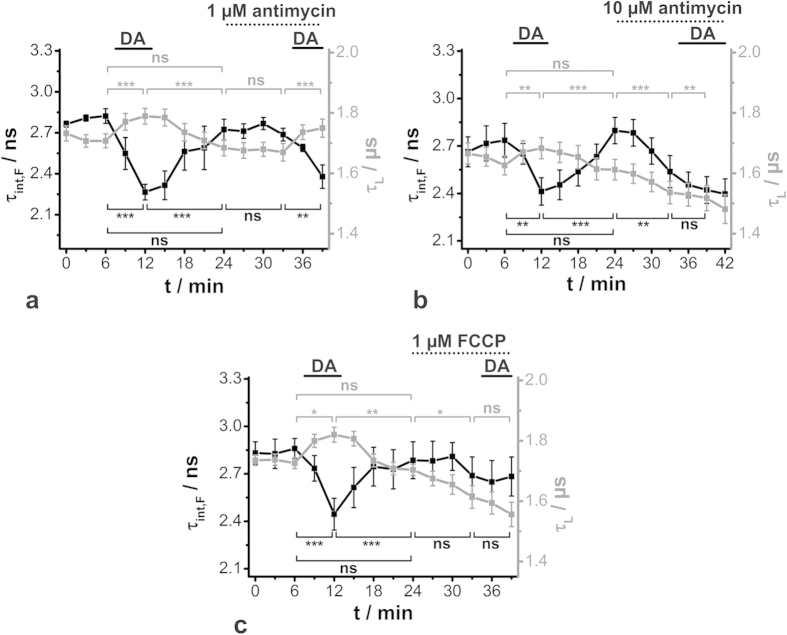
Effects of metabolic drugs on dopamine-induced (DA) changes of O_2_ and FAD in cockroach salivary duct cells by PLIM/FLIM recordings. τ_int,F_ (black, FAD) and τ_L_ (grey, O_2_) were extracted from PLIM/FLIM images (200 pixel × 200 pixel, 80 μm × 80 μm, pixel dwell time 2.0 ms/pixel, TCSPC time resolution 200 ps, repetition rate 50 kHz) and are shown as mean ± SEM and statistical analyses (***P < 0.001, **P < 0.01, *P < 0.05, ns: not significant). (**a**) Effect of 1 μM antimycin, *N* = 6. (**b**) Effect of 10 μM antimycin, *N* = 11 (**c**) Effect of 1 μM FCCP, *N* = 10.

Antimycin inhibits the electron transport chain, causing the same effect as cyanide, namely the stagnation of oxidative phosphorylation^43^,^44^. This normally leads to an increase in NADH fluorescence intensity, which implies an accumulation of reduced NADH and a concurrent decrease or slight increase in oxidized FAD fluorescence intensity^35^,^42^,^45^. A reduction in oxygen consumption can also be observed^46^,^47^. A control stimulus of isolated gland preparations with 1 μM dopamine at the beginning of an experiment resulted in the already unravelled opposing changes in τ_L_ and τ_int,F_, representing an elevated oxygen consumption and FAD protein binding (Fig. 7a). Subsequent bath application of 1 μM antimycin alone for 10 min did not alter τ_L_ and τ_int,F_. In addition, in 6 of the 12 preparations, a second dopamine stimulus in the presence of 1 μM antimycin still resulted in significant τ_L_ and τ_int,F_ changes, as observed for the control stimulus (Fig. 7a). In contrast, bath application of 10 μM antimycin alone induced statistically significant decreases in both τ_L_ and τ_int,F_. In addition, in the presence of 10 μM antimycin, the 1 μM dopamine-induced effects were altered dramatically, since no change in τ_int,F_ could be observed, but rather a further decrease in τ_L_ (Fig. 7b). The mitochondrial membrane uncoupler FCCP, on the other hand, abolishes the linkage between the electron transport chain and the phosphorylation system by extinguishing the established proton gradient^48^. In comparison to antimycin, the effects of FCCP are mostly the opposite, causing high levels of oxidized FAD and high oxygen consumption^35^,^42^,^46^,^47^. However, the results observed here did not clearly reflect that. Bath application of 1 μM FCCP induced a decrease in τ_L_, whereas τ_int,F_ was unchanged. Moreover, in the presence of FCCP, the dopamine effect was totally suppressed (Fig. 7c). Both antimycin and FCCP led to less oxygen consumption (decrease in *μs*-decay time) and less free oxidized FAD (decrease in *ns*-decay time), and both drugs could inhibit the increased metabolic activity due to dopamine stimulation. Although several studies of intracellular NADH, FAD and oxygen recordings have been reported, the underlying physiological and molecular mechanisms may be difficult to compare due to different cell types and microenvironments. Furthermore, comprehensive studies are required in order to correctly assign the measured luminescence intensities or decay times to fractions of free and protein-bound, as well as oxidized or reduced metabolic coenzymes^36^,^49^,^50^.

## Conclusions

In the present study, we proved the suitability of the novel TCSPC board TimeHarp 260 for simultaneous fluorescence/phosphorescence lifetime imaging microscopy (FLIM/PLIM) as the metabolic activity in insect salivary glands was investigated by recording the *ns*-decaying intrinsic cellular autofluorescence and *μs*-decaying phosphorescence of oxygen-sensitive Kr341.

With regard to biological applications, the concentration of analyte-sensitive dye always needs to be adjusted thoroughly. So, at extremely high sensor concentrations, toxic side effects are likely. However, the signal-to-noise ratio of FLIM/PLIM strongly depends on dye characteristics and concentration, respectively. To obtain an estimation of the necessary sensor concentrations, we start from the fact that confocal microscopes have a confocal volume in the *fL*-range. For *ns*-decaying sensor dyes with a high fluorescence quantum yield, single-molecule-sensitive setups have been available for a long time to study molecules one by one^51^,^52^,^53^. With a single molecule in the confocal volume, the sensor concentrations are normally in the *nM*-range. If a fluorophore in solution is excited by a laser with a repetition rate of 40 MHz, usually a detection count rate of at most a few 10^4^ photons/s and molecule can be achieved, depending on the dye, setup and laser intensity used. A dye with a 1,000-fold longer decay time in the *μs*-range would require a ~1,000-fold lower laser repetition rate. Therefore, the dye concentration has to be increased by the same factor in order to collect a similar amount of photons. Therefore, one can conclude that necessary concentrations for dyes with decay times in the *μs*-range are 1,000-fold higher, i.e. in the *μM*-range, in order to be measured with a similar acquisition time. For sensor dyes with even longer decay times, the necessary sensor concentrations have to be even higher.

On the other hand, if fluorescence and phosphorescence are to be monitored simultaneously, the laser repetition rate has to be drastically decreased in order to match the TCSPC window to the phosphorescent sensor. Here, an increase in the concentration of the fluorescent sensor a 1,000-fold will not result in a 1,000-fold increase in the fluorescence signal, as in the *ns*-range the dead time of the electronics still needs to be taken into consideration. With a single molecule in focus and a signal count rate of e.g. 10^3^ photons/s and molecule and a repetition rate of 40 MHz, a photon is detected about every 4,000th pulse (assuming no saturation conditions). This indicates that, for an excitation rate adapted to the phosphorescence dye, the concentration of the fluorescent dye has to be increased. However, in case of autofluorescence the concentration cannot be varied.

Nonetheless, we successfully applied the TCSPC board TimeHarp 260 for simultaneous recordings of *ns*-decaying intrinsic cellular autofluorescence and *μs*-decaying phosphorescence of an oxygen sensor by choosing an intermediate temporal resolution. The obtained decay time changes showed a similar trend compared to separately recorded fluorescence/phosphorescence data. In general, this FLIM/PLIM approach can also be applied in clinical studies. For instance, FLIM has been used to discriminate between healthy and cancerous tissue by analysing metabolic states^36^,^54^,^55^. In addition, in cancerous tissue, the oxygen concentration is often lower than in normal tissue, because of poor oxygen supply due to an impaired vascular network^56^. Therefore, FLIM/PLIM (two-parameter) mapping of tissues can provide a more reliable tissue analysis tool when studying human diseases.

Multiplexing concepts gain in importance, as they facilitate a comprehensive understanding of the spatial and temporal interactions between cellular parameters. We believe that the lifetime based-multiplexing approach is a promising complement to a well-established intensity-based multiparameter detection. However, luminescent sensor dyes with excellently separated decay times are needed, along with a TCSPC module with an appropriate time resolution^7^. Therefore, in general, any combination of organic fluorophores, fluorescent proteins or quantum dots can be applied to FLIM/PLIM, as long their luminescence decay times differ significantly.

Several ion-sensitive organic fluorescent dyes exhibit fluorescence decay times in the *ns*-range (0.1–10 ns) and have already been evaluated for FLIM recordings. Thereby, important physiological parameters such as intracellular concentration of Ca^2+^, Na^+^, Cl^–^ and pH were reliably quantified^57^,^58^,^59^,^60^,^61^.

In contrast, the variety of sensors with luminescence decay times from the long *ns*-range to the *ms*-range is limited. Quantum dots, for example, feature excellent photophysical properties, making them ideally suited for intracellular imaging^62^. Although quantum dots possess longer decay times than organic fluorophores (approx. 10–100 ns), their decay behaviour is rather multiexponential, which might cause difficulties when a multicomponent luminescence decay of several sensors is analysed. Moreover, up to now, the combination of FLIM and quantum dots for intracellular sensing has been reported in the literature only once. There, the intracellular pH of living cells could be determined by analysing the decay time changes of quantum dots within a range of 8–16 ns^63^. However, it can be predicted that the chemical modification of quantum dot surfaces will generate new intracellular quantum dot-based sensors with longer decay times in the future^64^,^65^.

On the other hand, complexes of lanthanides such as Eu^3+^ and Tb^3+^ also exhibit very long luminescence decay times within the *μs-ms*-range^66^. In principle, they are ideally suited for studying molecular interactions *via* luminescence resonance energy transfer (LRET), but they have mainly been used in *in vitro* measurements^67^. Recently, a lanthanide-based complex for *in vivo* luminescence imaging was developed^68^,^69^. However, limitations of lanthanide complexes such as stability, toxicity and intracellular uptake will require substantial research progress.

In summary, FLIM/PLIM is a useful multiplexing strategy, and with further improvements of adequate sensors, it will significantly contribute towards unravelling cellular functions.

## Methods

### Chemicals and solutions

The O_2_-sensitive dye Kr341 (Bis[(1,10-phenanthroline-4,7-diyl-κ*N*,κ*N*’)bis(phenylsulfonate)(2-)][*N*-octadecyl-5-(4-(7-phenyl-1,10-phenanthroline-4-yl-κ*N*,κ*N*’)phenyl)pentanamide] ruthenium (II) dichloride) was provided by the group of W. Bannwarth (University of Freiburg)^18^. Kr341 was dissolved in dimethyl sulfoxide (DMSO) to receive a 2.59 mM stock solution. The absorption and luminescence emission spectra of Kr341 were recorded in aqueous solution containing 20 μM Kr341. Absorption and fluorescence emission spectra of flavin adenine dinucleotide disodium salt hydrate FAD (Sigma-Aldrich, Deisenhofen, Germany) were recorded in phosphate buffered saline (PBS) (pH 7.0) containing 9 μM FAD.

Physiological saline contained 160 mM NaCl, 10 mM KCl, 2 mM CaCl_2_, 2 mM MgCl_2_, 10 mM glucose, 10 mM Tris and 100 μM DABCO (Sigma-Aldrich, Deisenhofen, Germany). The pH was adjusted to pH 7.4 with HCl. To convert the measured luminescence decay times to O_2_ concentration [O_2_], a three-point calibration procedure was carried out. Therefore, physiological saline was flushed with N_2_, ambient atmosphere or O_2_ for at least 30 min before starting a measurement obtaining [O_2_] values of 1289 μM, 254 μM and 0 μM (T = 299.15 K). The oxygen concentrations were determined from literature data by applying Henry’s law^70^,^71^. During data acquisition, the reservoir containing the physiological saline was continuously flushed with adequate gas.

A stock solution of 10 mM dopamine (Sigma-Aldrich, Deisenhofen, Germany) was prepared daily in double-distilled water and was diluted in physiological saline immediately before an experiment in order to obtain a final concentration of 1 μM dopamine. Stock solutions of antimycin and carbonyl cyanide-*4*-(trifluoromethoxy)phenylhydrazone (FCCP) (both Sigma-Aldrich, Deisenhofen, Germany) were dissolved in DMSO and stored at −20 °C. Immediately before an experiment, antimycin and FCCP were diluted in physiological saline to obtain final concentrations of 1 μM FCCP, 1 μM and 10 μM antimycin, respectively.

### Absorption and luminescence spectra

Absorption measurements were performed with a Lambda 750 UV/VIS spectrometer (Perkin Elmer, Waltham, USA) in the range of 250 nm <λ_ex_ < 700 nm. Luminescence emission spectra (450 nm <λ_em_ < 700 nm, spectral bandwidth Δλ = 1 nm) were recorded with a FluoroMax 4 (Horiba, Kyoto, Japan). Concentrations were adjusted to avoid inner filter effects (absorption maximum below 0.1).

### Tissue preparation

A colony of the American cockroaches *Periplaneta americana* was reared at 27 ^o^C under a light/dark cycle of 12 h : 12 h at the Department of Animal Physiology (University of Potsdam). The animals had free access to food and water. Only male adults aged 4–6 weeks were used for experiments. Salivary glands were dissected in physiological saline as described previously^57^. Small lobes consisting of several acini with their corresponding branched duct system were examined. The gland lobes were incubated in either physiological saline or physiological saline containing 38 μM Kr341 for 30 min at room temperature and were subsequently acclimatised for 10 min in fresh physiological saline. Then, lobes were attached to a glass coverslip using the tissue adhesive Vectabond (Axxora, Lörrach, Germany). The perfusion chamber was mounted on the microscope stage, and during data acquisition the glands were continuously perfused with physiological saline.

### FLIM and PLIM recordings

**Figure 8:**
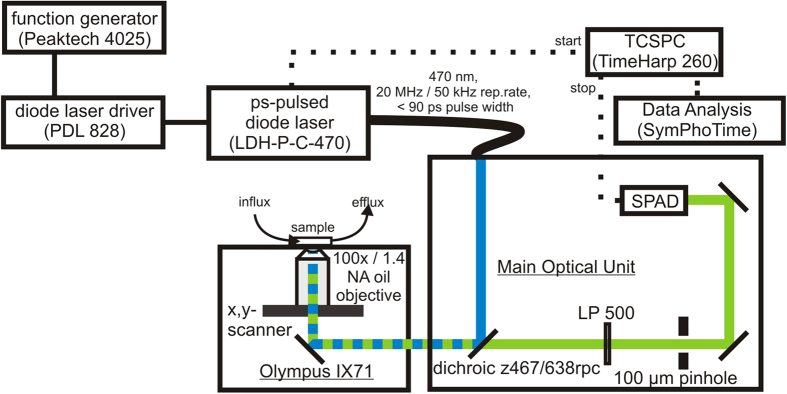
Experimental setup. Scheme of the setup for FLIM and PLIM, based on the MicroTime 200 fluorescence lifetime microscope system. For details, see text.

For time-resolved luminescence imaging, a MicroTime 200 time-resolved confocal fluorescence microscope (PicoQuant, Berlin, Germany) was used (Fig. 8), consisting of an inverted microscope (IX 71, Olympus) equipped with an Olympus PlanApo 100 × /NA 1.4 oil immersion objective. A ps-pulsed diode laser (LDH-P-C-470, PicoQuant, Berlin, Germany) operating at 470 nm with a pulse width of <90 ps was used as an excitation source. The repetition rate could be adjusted either by a function generator (Peaktech 4025, Peaktech, Ahrensburg, Germany, *f*_rep_ = 50 kHz) or directly by the diode laser driver (PDL 828, PicoQuant, Berlin, Germany, *f*_rep_ set to 20 MHz). The laser beam was guided towards the objective using the dichroic mirror z467/638 rpc (AHF Analysentechnik, Germany). The luminescence was guided through a 100 μm pinhole and was detected with a single-photon avalanche diode (SPAD, SPCM-AQR-13, Perkin Elmer, Waltham, USA). For the separation of luminescence and rejection of the excitation light, a long-pass filter LP500 was used (AHF Analysentechnik, Germany). Time-resolved luminescence recordings were performed in the time-correlated single-photon counting (TCSPC) mode using a TimeHarp 260 PICO board (PicoQuant, Berlin, Germany), which features a broad time channel resolution range of 25 ps–52 μs combined with a multi-stop functionality. In all experiments, the laser power was adjusted to achieve average photon counting rates ≤10^5^ photons/s and peak rates close to 10^6^ photons/s when recording images, thus significantly below the maximum counting rates allowed by TCSPC electronics in order to avoid pile up effects. Images were acquired by raster scanning the objective using a *xy*-piezo positioner (Physik Instrumente, Karlsruhe, Germany). Data acquisition and analysis were performed by the SymPhoTime64 software version 1.5 (PicoQuant, Berlin, Germany). Thereby, all photons collected in the full frame image were used to form a global histogram for luminescence decay fitting. For deconvolution fitting, the instrument response function (IRF) was measured daily by recording the backscattered excitation light. Its full width at half-maximum (FWHM) was calculated to 530 ± 20 ps (means ± SEM, *N* = 7). A high quality of luminescence decay fitting was estimated by means of randomly distributed residuals and low reduced 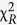 values.

### Data analyses

The recorded data were distributed normally (D’Agostino-Pearson normality test for *N* > 6; Kolmogorov-Smirnov test for *N* = 6). Thus, the data set was further analysed by repeated-measures ANOVA and subsequent Holm-Sidak’s multiple comparisons tests of selected data pairs. Here, within the figures the used data set for one data pair analysis was marked by the endings of a corresponding bracket including the significance level. In pharmacological experiments, only recordings with positive dopamine control stimuli were analysed. Differences were considered statistically significant if *P* < 0.05. Statistical analyses were performed using GraphPad Prism (GraphPad Software, San Diego, USA). The graphical presentation was carried out with Origin (OriginLab, Northampton, USA). The data were presented as means ± standard error of the mean (SEM).

## Additional Information

**How to cite this article**: Jahn, K. *et al.* Simultaneous Fluorescence and Phosphorescence Lifetime Imaging Microscopy in Living Cells. *Sci. Rep.* **5**, 14334; doi: 10.1038/srep14334 (2015).

## Supplementary Material

Supplementary Information
