# Supplementary material for: Simultaneous Fluorescence and Phosphorescence Lifetime Imaging Microscopy in Living Cells
Source: Sci Rep. 2015 Sep 22;5:14334. doi: 10.1038/srep14334 (PMC4585718; doi:10.1038/srep14334)
Supplement: Supplementary Information [file srep14334-s1.pdf]

Supplementary Information

Simultaneous Fluorescence and Phosphorescence Lifetime Imaging Microscopy in Living Cells

Karolina Jahn[1], Volker Buschmann[2], Carsten Hille[1,*]

[1] Physical Chemistry / ALS ComBi, Institute of Chemistry, University of Potsdam, Potsdam, Germany

[2] PicoQuant GmbH, Berlin, Germany

* *Address for correspondence*:

Carsten Hille, Physical Chemistry / ALS ComBi, Institute of Chemistry, University of Potsdam, Karl-Liebknecht-Str. 24-25, 14476 Potsdam, Germany

E-mail: hille@uni-potsdam.de; Tel.: +49-331-977 5255; Fax: +49-331-977 6137

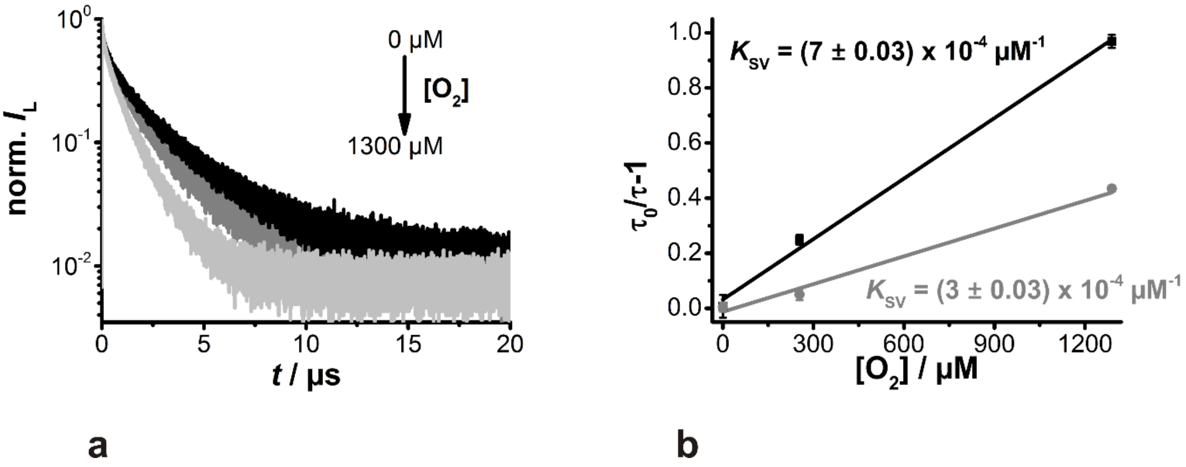

Supplementary Figure S1 Oxygen-responsive Kr341 quenching experiments. (a) *In vitro* luminescence decay curves of Kr341 in the presence of 0 µM (black), 254 µM (dark grey) and 1289 µM (grey) oxygen (T = 299.15°K). Variation of the oxygen concentration from 0 µM to 1289 µM reduced the *in vitro* luminescence decay time of Kr341 from (2.08 ± 0.05) µs to (1.06 ± 0.01) µs (mean ± SEM, *N* = 5). (b) Stern-Volmer plots of *in vitro* and *in situ* time-resolved luminescence measurements. The linear fit to the data points yielded an *in vitro* K_{SV} = (7 ± 0.3) × 10^{-4} μM^{-1} (R^2 = 0.975) and an *in situ* K_{SV} = (3 ± 0.3) × 10^{-4} μM^{-1} (R^2 = 0.786), respectively (mean ± SEM, *N* = 5-17). Variation of the oxygen concentration from 0 µM to 1289 µM reduced the *in situ* luminescence decay time of Kr341 from (2.44 ± 0.04) µs to (1.70 ± 0.01) µs (mean ± SEM, *N* = 5-17).

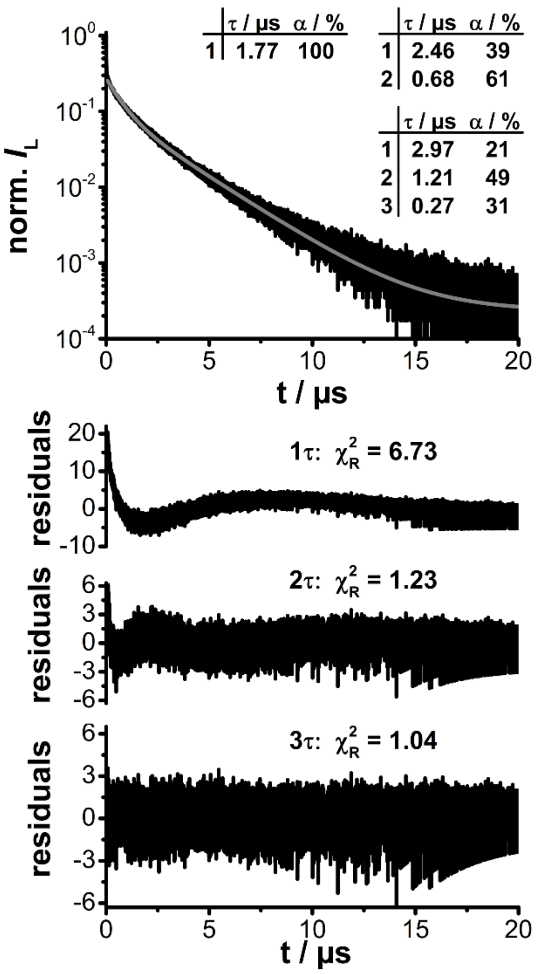

Supplementary Figure S2 Time-resolved luminescence recordings of Kr341-loaded salivary duct cells. Luminescence decay curve (black) extracted from a PLIM image of Kr341-loaded salivary duct cells and the corresponding biexponential tail-fit (grey). For comparison, the decay time components with their normalised amplitudes of mono-, bi- and triexponential fit models are shown (inset) and their weighted residuals and corresponding χ^2_R values are displayed below.

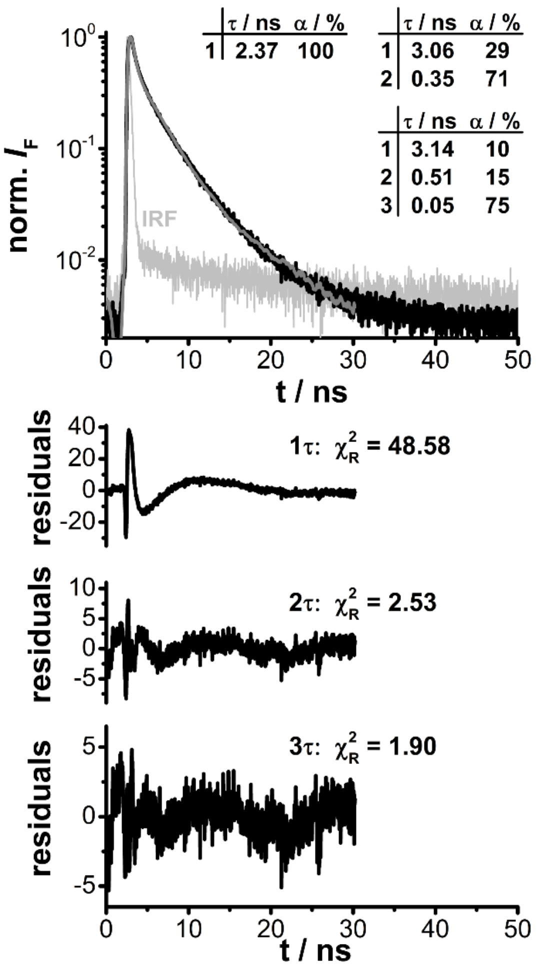

Supplementary Figure S3 Time-resolved autofluorescence recordings of salivary duct cells. Fluorescence decay curve (black) extracted from a FLIM image of untreated salivary duct cells with corresponding instrument response function (IRF) and biexponential deconvolution fit (grey). For comparison, the decay time components with their normalised amplitudes of mono-, bi- and triexponential fit models are shown (inset) and their weighted residuals and corresponding χ_R^2 values are displayed below.

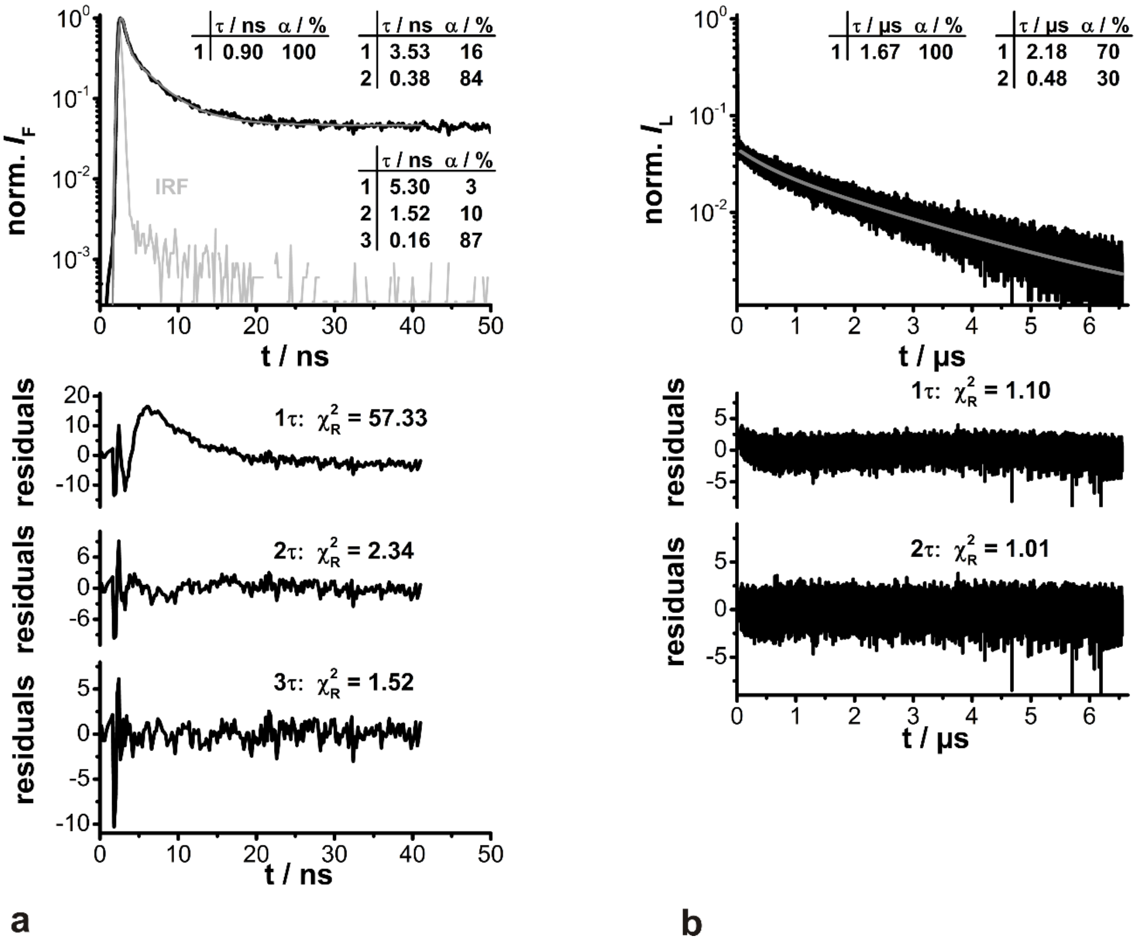

Supplementary Figure S4 Simultaneous time-resolved autofluorescence / luminescence recording of Kr341-loaded salivary duct cells. (a) Fluorescence decay curve (black) extracted from a FLIM/PLIM image of salivary duct cells with corresponding instrument response function (IRF) and biexponential deconvolution fit (grey). For comparison, the decay time components with their normalised amplitudes of mono-, bi- and triexponential fit models are shown (inset) and their weighted residuals and corresponding χ_R^2 values are displayed below. (b) Luminescence decay curve extracted from the FLIM/PLIM image analysed in (a) of Kr341-loaded salivary duct cells and the corresponding monoexponential tail-fit (grey). For comparison, the decay time components with their normalised amplitudes of mono - and biexponential fit models are shown (inset) and their weighted residuals and corresponding χ_R^2 values are displayed below.

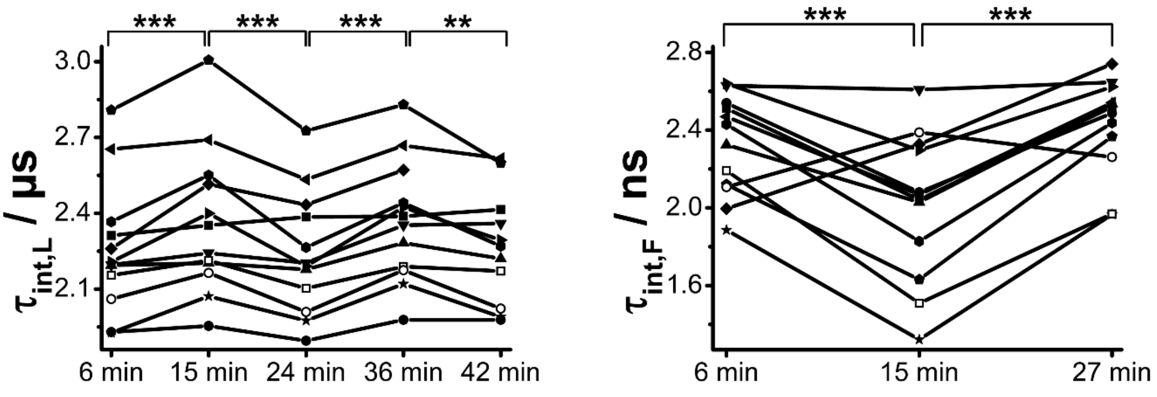

a **b**

Supplementary Figure S5 Individual variations of PLIM and FLIM recordings in different isolated salivary gland preparations. (a) 12 individual measurements of the dopamine-induced changes in Kr341 luminescence decay time $\tau_{int,L}$ in Kr341-treated salivary duct cells and statistical analyses (***P < 0.001, **P < 0.01). Means ± SEM are shown in Fig. 2b. 1 µM dopamine is present at 15. min and 36. min. (b) 12 individual measurements of dopamine-induced changes in FAD fluorescence decay time $\tau_{nt,F}$ in salivary duct cells and statistical analyses (***P < 0.001). Means ± SEM are shown in Fig. 5a. 1 µM dopamine is present at 15. min.
